# Supplementary material for: Investigating Whether the Mediterranean Dietary Pattern Is Integrated in Routine Dietetic Practice for Management of Chronic Conditions: A National Survey of Dietitians
Source: Nutrients. 2020 Nov 4;12(11):3395. doi: 10.3390/nu12113395 (PMC7694348; doi:10.3390/nu12113395)
Supplement: Supplementary file 1 [file nutrients-12-03395-s001.pdf]

SUPPLEMENTARY MATERIAL

Investigating whether the Mediterranean dietary pattern is integrated in routine dietetic practice for management of chronic conditions: A national survey of dietitians

Hannah L Mayr*, Sarah P Kostjasyn*, Katrina L Campbell, Michelle Palmer, Ingrid J Hickman

*Joint first author.

Correspondence to: Dr Hannah L Mayr, Nutrition and Dietetics Department, Building 15, Princess Alexandra Hospital, 199 Ipswich Rd, Woolloongabba, Queensland 4102, Australia. hmayr@bond.edu.au; ph +61 7 3176 7938

Table S1. Dietitian participants Australian State or Territory of work

State or Territory	*n (%)*
New South Wales	42 (23)
Victoria	46 (25)
Queensland	58 (32)
South Australia	6 (3)
Northern Territory	2 (1)
Western Australia	21 (12)
Tasmania	5 (3)
Australian Capital Territory	2 (1)

Table S2. Statistical comparisons between how often dietitians counsel chronic disease populations on Mediterranean dietary pattern and characteristics of their relevant practice

	**Cardiovascular disease**			**Type 2 diabetes**			**Non-alcoholic fatty liver disease**		
How often counsel MDP	Never/ rarely/ sometimes	Most of the time/ always	P	Never /rarely/ sometimes	Most of the time/ always	P	Never/ rarely/ sometimes	Most of the time/ always	P
Relevant practice experience									
0-5 years	43 (66)	22 (34)	**0.005**	61 (88)	8 (12)	**0.001**	37 (84)	7 (16)	**0.01**
6+ years	41 (42)	56 (58)		68 (64)	38 (36)		47 (60)	31 (40)	
Relevant work location									
Metropolitan	52 (51)	51 (49)	0.77	79 (69)	35 (31)	0.10	47 (62)	29 (38)	0.05
Regional or rural	32 (54)	27 (46)		50 (82)	11 (18)		37 (80)	9 (20)	
Patient setting									
Acute or subacute	28 (72)	11 (28)	**0.007**	37 (88)	5 (12)	**0.03**	17 (68)	8 (32)	1.00
Community or outpatient	56 (46)	67 (56)		92 (69)	41 (31)		67 (69)	30 (31)	
Healthcare setting									
Public hospital or community service	49 (55)	40 (45)	0.52	79 (78)	22 (22)	0.32	45 (69)	20 (31)	0.81
Private hospital or community practice	32 (48)	34 (52)		47 (70)	20 (30)		34 (65)	18 (35)	
Personally follow MDP									
Never/ rarely/ sometimes	46 (73)	17 (27)	**<0.001**	63 (91)	6 (9)	**<0.001**	37 (77)	11 (23)	0.17
Most of the time/ always	37 (39)	58 (61)		63 (62)	39 (38)		45 (63)	26 (37)	
Knowledgeable of MDP									
Strongly disagree/ disagree/ neither	5 (63)	3 (37)	**<0.001**	6 (75)	2 (25)	**<0.001**	5 (83)	1 (17)	**0.001**
Somewhat agree	56 (75)	19 (25)		76 (91)	9 (10)		46 (85)	8 (15)	
Strongly agree	23 (29)	56 (71)		47 (57)	36 (43)		33 (53)	29 (47)	
Confident to counsel patient on MDP									
Strongly disagree/ disagree/ neither	15 (83)	3 (17)	**<0.001**	18 (95)	1 (5)	**<0.001**	10 (100)	0	**<0.001**
Somewhat agree	46 (70)	20 (30)		66 (87)	10 (13)		44 (86)	7 (14)	
Strongly agree	23 (30)	55 (70)		45 (56)	35 (44)		30 (49)	31 (51)	
Recommending MDP part of role									
Strongly disagree/ disagree/ neither	35 (92)	3 (8)	**<0.001**	44 (98)	1 (2)	**<0.001**	29 (98)	1 (3)	**<0.001**
Somewhat agree	36 (50)	36 (50)		58 (74)	20 (26)		36 (77)	11 (23)	
Strongly agree	13 (25)	39 (75)		27 (52)	25 (48)		19 (42)	26 (58)	

Data are n (%). Total counts for each comparison are reflective of the number of participants with relevant responses to both survey questions. Significant differences across categories **p<0.05**

Table S3. Statistical comparisons between dietitians perceived knowledge, confidence to counsel and role regarding Mediterranean dietary pattern (MDP) and characteristics of their relevant practice

	**Knowledgeable of MDP**				**Confident to counsel patient on MDP**				**Recommending MDP part of role**			
Level of agreement	Disagree / neither	Somewhat agree	Strongly agree	P	Disagree / neither	Somewhat agree	Strongly agree	P	Disagree / neither	Somewhat agree	Strongly agree	P
Relevant practice experience												
0-5 years	5 (7)	37 (51)	31 (43)	0.38	12 (16)	31 (43)	30 (42)	0.14	22 (30)	38 (52)	13 (18)	**0.02**
6+ years	4 (4)	49 (45)	56 (51)		8 (7)	47 (43)	54 (50)		25 (23)	44 (40)	40 (37)	
Relevant work location												
Metropolitan	7 (6)	49 (41)	64 (53)	0.05	11 (9)	46 (38)	63 (53)	0.05	33 (28)	48 (40)	39 (32)	0.15
Regional or rural	2 (3)	37 (60)	23 (37)		9 (15)	32 (52)	21 (44)		14 (23)	34 (54)	14 (23)	
Patient setting												
Acute or subacute	2 (4)	28 (61)	16 (35)	0.10	5 (11)	25 (54)	16 (35)	0.16	17 (37)	20 (44)	9 (20)	0.09
Community or outpatient	7 (5)	58 (43)	71 (52)		15 (11)	53 (39)	68 (50)		30 (22)	62 (46)	44 (32)	
Healthcare setting												
Public hospital or community service	7 (7)	53 (50)	47 (44)	0.24	14 (13)	53 (50)	40 (37)	**0.01**	31 (29)	55 (51)	21 (20)	**0.002**
Private hospital or community practice	2 (3)	28 (41)	38 (56)		12 (7)	22 (32)	41 (60)		15 (22)	23 (34)	30 (44)	
Personally follow MDP												
Never/ rarely/ sometimes	5 (7)	46 (64)	21 (29)	**<0.001**	10 (14)	38 (53)	24 (33)	**0.01**	27 (38)	35 (49)	10 (14)	**<0.001**
Most of the time / always	4 (4)	37 (35)	65 (61)		10 (9)	37 (35)	59 (56)		20 (19)	44 (41)	42 (40)	

Data are n (%). Total counts for each comparison are reflective of the number of participants with relevant responses to both survey questions. Significant differences across categories **p<0.05**

Document S1. National Survey questions including conditional release description

^Q1 To enter the survey, please click the arrow at the bottom right of this page.

Q2 Which of the following patient groups have you recently (within the past 12 months) practiced with as a dietitian in Australia? *Tick all that apply*

- ○ Cardiovascular disease
- ○ Type 2 Diabetes
- ○ Non-alcoholic fatty liver disease (NAFLD)
- ○ Solid organ transplant recipients (not including acute care only)
- ○ None of these patient groups

Skip To: End of Survey If Q2 = None of these patient groups

Display This Question:
If Q2 = Cardiovascular disease, Type 2 Diabetes, Non-alcoholic fatty liver disease (NAFLD), or Solid organ transplant recipients (not including acute care only)

Q3 You are eligible to continue this survey. Please note that the questions asked are intended to relate to your current or recent work as a dietitian with the relevant patient group/s you identified in the previous question. This will be referred to in the survey as 'your relevant dietitian role'.

Q4 What is your age? *Please enter years*

Q5 What is your gender?

- ○ Male
- ○ Female
- ○ Other
- ○ Prefer not to answer

Q6 Were you born in Australia?

- ○ Yes
- ○ No

Display This Question:
If Q6 = No

Q7 What country were you born in? *Please specify*

Q8 Were either of your parents born outside of Australia?

- ◯ Yes
- ◯ No

Display This Question:
If Q8 = Yes

Q9 What country / countries were they born in? *Please specify*

Q10 In which Australian State do you work (or did you work) in your relevant dietitian role?

- ◯ New South Wales
- ◯ Victoria
- ◯ Queensland
- ◯ South Australia
- ◯ Northern Territory
- ◯ Western Australia
- ◯ Tasmania
- ◯ Australian Capital Territory

Q11 In your relevant dietitian role, what is or was the main location of your work?

- ◯ Metropolitan
- ◯ Regional
- ◯ Rural
- ◯ Other, please specify:

Q12 Please enter the postcode of the main place of your relevant dietitian role.
This question is optional if you do not wish to answer

Q13 In your relevant dietitian role, what is or was your main place of work?

- ○ Public Hospital
- ○ Private Hospital
- ○ Public Community Health Service
- ○ Private Practice
- ○ Aged Care
- ○ University
- ○ Corporate Health
- ○ Other, please specify:

Q14 What year did you graduate from your dietetics degree? *Please specify year*

Q15 In your career as a dietitian, how long would you estimate is the total time you have been working with chronic disease patients with either cardiovascular disease, type 2 diabetes, NAFLD, or solid organ transplant recipients?

- ○ < 1 year
- ○ 1 to 2 years
- ○ 3 to 5 years
- ○ 6 to 10 years
- ○ 11 to 15 years
- ○ 16 to 20 years
- ○ 20 or more years

Q16 In your relevant dietitian role, what is or was the <u>main</u> patient setting of the service?

- ○ Acute
- ○ Sub-acute
- ○ Outpatient
- ○ Community
- ○ Other, please specify:

Display This Question:
If Q2 = Cardiovascular disease

Q17 You identified that you have recently treated patients with cardiovascular disease, which conditions does this include? *Tick all that apply*

- ◯ Coronary heart disease / acute coronary syndrome
- ◯ Hypertension
- ◯ Dyslipidaemia
- ◯ Heart failure
- ◯ Other *Please specify*

Display This Question:
If Q2 = Solid organ transplant recipients (not including acute care only)

Q18 You identified that you have recently treated solid organ transplant recipients, which of the following does that include? *Tick all that apply*

- ◯ Liver transplant
- ◯ Kidney transplant
- ◯ Heart transplant
- ◯ Other *Please specify*

^Q19 Please select whether you agree or disagree with the following statement/s related to the patient groups you identified working with

Display This Question:
If Q2 = Cardiovascular disease

Q20 There is enough evidence to support recommending the Mediterranean diet pattern to my patients with cardiovascular disease

- ◯ Strongly disagree
- ◯ Somewhat disagree
- ◯ Neither agree nor disagree
- ◯ Somewhat agree
- ◯ Strongly agree

Display This Question:
If Q2 = Type 2 Diabetes

Q21 There is enough evidence to support recommending the Mediterranean diet pattern to my patients with type 2 diabetes

- ◯ Strongly disagree
- ◯ Somewhat disagree
- ◯ Neither agree nor disagree
- ◯ Somewhat agree
- ◯ Strongly agree

Display This Question:
If Q2 = Non-alcoholic fatty liver disease (NAFLD)

Q22 There is enough evidence to support recommending the Mediterranean diet pattern to my patients with NAFLD

- ◯ Strongly disagree
- ◯ Somewhat disagree
- ◯ Neither agree nor disagree
- ◯ Somewhat agree
- ◯ Strongly agree

Display This Question:
If Q18 = Liver transplant

Q23 There is enough evidence to support recommending the Mediterranean diet pattern (long term) to my patients who have received a liver transplant

- ◯ Strongly disagree
- ◯ Somewhat disagree
- ◯ Neither agree nor disagree
- ◯ Somewhat agree
- ◯ Strongly agree

Display This Question:
If Q18 = Kidney transplant

Q24 There is enough evidence to support recommending the Mediterranean diet pattern (long term) to my patients who have received a kidney transplant

- ◯ Strongly disagree
- ◯ Somewhat disagree
- ◯ Neither agree nor disagree
- ◯ Somewhat agree
- ◯ Strongly agree

Display This Question:
If Q18 = Heart transplant

Q25 There is enough evidence to support recommending the Mediterranean diet pattern (long term) to my patients who have received a heart transplant

- ◯ Strongly disagree
- ◯ Somewhat disagree
- ◯ Neither agree nor disagree
- ◯ Somewhat agree
- ◯ Strongly agree

Display This Question:
If Q18 = Other *Please specify*

Q26 There is enough evidence to support recommending the Mediterranean diet pattern (long term) to my patients who have received a solid organ transplant

- ◯ Strongly disagree
- ◯ Somewhat disagree
- ◯ Neither agree nor disagree
- ◯ Somewhat agree
- ◯ Strongly agree

Q27 Do you have any additional comments in relation to evidence for the Mediterranean diet pattern for relevant chronic conditions? *Please specify*

^Q28 Please select whether you agree or disagree with the following statements

Q29 I am knowledgeable of the principles of the Mediterranean diet pattern

- ◯ Strongly disagree
- ◯ Somewhat disagree
- ◯ Neither agree nor disagree
- ◯ Somewhat agree
- ◯ Strongly agree

Q30 I am confident to describe to a colleague the key principles of the Mediterranean diet pattern

- ◯ Strongly disagree
- ◯ Somewhat disagree
- ◯ Neither agree nor disagree
- ◯ Somewhat agree
- ◯ Strongly agree

Q31 I am confident to counsel a patient to follow the principles of the Mediterranean diet pattern

- ◯ Strongly disagree
- ◯ Somewhat disagree
- ◯ Neither agree nor disagree
- ◯ Somewhat agree
- ◯ Strongly agree

Q32 Recommending the Mediterranean diet to patients with chronic disease is part of my role

- ◯ Strongly disagree
- ◯ Somewhat disagree
- ◯ Neither agree nor disagree
- ◯ Somewhat agree
- ◯ Strongly agree

Q33 Do you have any additional comments in relation to these questions related to your knowledge, confidence, and role? *Please specify*

Display This Question:
If Q2 = Cardiovascular disease

Q34 When you see patients with cardiovascular disease how often do you counsel on the Mediterranean diet?

- Never
- Rarely
- Sometimes
- Most of the time
- Always

Display This Question:
If Q2 = Type 2 Diabetes

Q35 When you see patients with type 2 diabetes how often do you counsel on the Mediterranean diet?

- Never
- Rarely
- Sometimes
- Most of the time
- Always

Display This Question:
If Q2 = Non-alcoholic fatty liver disease (NAFLD)

Q36 When you see patients with NAFLD how often do you counsel on the Mediterranean diet?

- Never
- Rarely
- Sometimes
- Most of the time
- Always

Display This Question:
If Q18 = Liver transplant

Q37 When you see patients who are liver transplant recipients (excluding acute care) how often do you counsel on the Mediterranean diet?

- Never
- Rarely
- Sometimes
- Most of the time
- Always

Display This Question:
If Q18 = Kidney transplant

Q38 When you see patients who are kidney transplant recipients (excluding acute care) how often do you counsel on the Mediterranean diet?

- Never
- Rarely
- Sometimes
- Most of the time
- Always

Display This Question:
If Q18 = Heart transplant

Q39 When you see patients who are heart transplant recipients (excluding acute care) how often do you counsel on the Mediterranean diet?

- Never
- Rarely
- Sometimes
- Most of the time
- Always

Display This Question:
If Q18 = Other *Please specify*

Q40 When you see patients who are solid organ transplant recipients (excluding acute care) how often do you counsel on the Mediterranean diet?

- Never
- Rarely
- Sometimes
- Most of the time
- Always

Display This Question:
If any of Q34 to Q40 = Sometimes, Most of the time or Always

Q41 How long has the Mediterranean diet been incorporated (at least some of the time) in your practice?

- <6 months
- 6 months to 1 year
- 1 to 2 years
- 3 to 5 years
- More than 5 years

Q42 How often do other dietitians that you work with or know recommend the Mediterranean diet to patients with chronic disease?

- Never
- Rarely
- Sometimes
- Most of the time
- Always
- I don't know

^Q43 Please select the most appropriate option for the following questions:

How often do you incorporate the following dietary recommendations in your advice, education or resources to the relevant chronic disease patient group/s.
Consider serving size as reflecting the Australian Dietary Guidelines.

Q44 Daily intake of fruit (2-3 serves)

- ◯ Never
- ◯ Rarely
- ◯ Sometimes
- ◯ Most of the time
- ◯ Always

Q45 Daily intake of vegetables (5 or more serves)

- ◯ Never
- ◯ Rarely
- ◯ Sometimes
- ◯ Most of the time
- ◯ Always

Q46 Daily intake of tomatoes

- ◯ Never
- ◯ Rarely
- ◯ Sometimes
- ◯ Most of the time
- ◯ Always

Q47 Daily intake of leafy green salads/vegetables

- ◯ Never
- ◯ Rarely
- ◯ Sometimes
- ◯ Most of the time
- ◯ Always

Q48 Regular use of onion and garlic in cooking

- ○ Never
- ○ Rarely
- ○ Sometimes
- ○ Most of the time
- ○ Always

Q49 Regular use of herbs and spices in cooking

- ○ Never
- ○ Rarely
- ○ Sometimes
- ○ Most of the time
- ○ Always

Q50 Daily intake of wholegrain cereals (6-8 serves)

- ○ Never
- ○ Rarely
- ○ Sometimes
- ○ Most of the time
- ○ Always

Q51 Use of extra virgin olive oil as the main dietary fat

- ○ Never
- ○ Rarely
- ○ Sometimes
- ○ Most of the time
- ○ Always

Q52 Daily intake of extra virgin olive oil 3-4 tablespoons per day

- ◯ Never
- ◯ Rarely
- ◯ Sometimes
- ◯ Most of the time
- ◯ Always

Q53 Intake of fermented dairy foods (yoghurt or cheese) on most days

- ◯ Never
- ◯ Rarely
- ◯ Sometimes
- ◯ Most of the time
- ◯ Always

Q54 Limit intake of red meat to no more than 1 serve per week

- ◯ Never
- ◯ Rarely
- ◯ Sometimes
- ◯ Most of the time
- ◯ Always

Q55 Limit intake of processed/deli meats and small goods

- ◯ Never
- ◯ Rarely
- ◯ Sometimes
- ◯ Most of the time
- ◯ Always

Q56 Regular intake of legumes/lentils (2 or more serves per week)

- Never
- Rarely
- Sometimes
- Most of the time
- Always

Q57 Regular intake of fish/seafood (3 or more serves per week)

- Never
- Rarely
- Sometimes
- Most of the time
- Always

Q58 Intake of nuts on most days

- Never
- Rarely
- Sometimes
- Most of the time
- Always

Q59 If choosing to drink alcohol, moderate consumption of wine (1-2 glasses per day) with meals

- Never
- Rarely
- Sometimes
- Most of the time
- Always

Q60 Limit intake of sugary drinks

- Never
- Rarely
- Sometimes
- Most of the time
- Always

Q61 Limit intake of commercial or processed sweets/goods/savoury snacks

- Never
- Rarely
- Sometimes
- Most of the time
- Always

Q62 Encourage eating with others or shared meals

- Never
- Rarely
- Sometimes
- Most of the time
- Always

Q63 Encourage home cooking

- Never
- Rarely
- Sometimes
- Most of the time
- Always

Q64 Do you have additional comments in relation to these questions on how often you counsel on the Mediterranean diet or is there anything else you usually recommend in relation to Mediterranean diet? *Please specify*

Q65 Considering the principles which were previously described, would you estimate that you personally follow a Mediterranean diet pattern:

- ○ Never
- ○ Rarely
- ○ Sometimes
- ○ Most of the time
- ○ Always

Q66 Have you received any education or undertaken training or self-study on the Mediterranean diet pattern?

- ○ Yes
- ○ No

Display This Question:
If Q66 = Yes

Q67 What was this education, training, or self-study? *Tick all that apply*

- ○ Dietetics degree
- ○ Professional development provided at work
- ○ Professional development accessed outside of work (not a conference)
- ○ Reading scientific literature
- ○ Research activities
- ○ Australian conference
- ○ International conference
- ○ Other, please specify:

Display This Question:
If Q66 = Yes

Q68 Can you please provide further comment on whether this was useful or not and why? *Please comment/ explain:*

^Q69 For each of the following, please select whether you think they are a barrier or an enabler to whether <u>you</u> recommend the Mediterranean diet pattern to your relevant patients with chronic disease

Q70 Access to or awareness of relevant evidence / practice guidelines

- ◯ More often a barrier
- ◯ More often an enabler
- ◯ Both a barrier and an enabler
- ◯ Neither a barrier or an enabler
- ◯ Not relevant to my practice

Q71 Access to or awareness of professional development, education, or training for dietitians

- ◯ More often a barrier
- ◯ More often an enabler
- ◯ Both a barrier and an enabler
- ◯ Neither a barrier or an enabler
- ◯ Not relevant to my practice

Q72 Access to or awareness of relevant patient education materials or resources

- ◯ More often a barrier
- ◯ More often an enabler
- ◯ Both a barrier and an enabler
- ◯ Neither a barrier or an enabler
- ◯ Not relevant to my practice

Q73 Acceptability of the diet principles by patients

- ◯ More often a barrier
- ◯ More often an enabler
- ◯ Both a barrier and an enabler
- ◯ Neither a barrier or an enabler
- ◯ Not relevant to my practice

Q74 Goals or motivation of patients in relation to diet

- More often a barrier
- More often an enabler
- Both a barrier and an enabler
- Neither a barrier or an enabler
- Not relevant to my practice

Q75 Your own cultural background

- More often a barrier
- More often an enabler
- Both a barrier and an enabler
- Neither a barrier or an enabler
- Not relevant to my practice

Q76 Cultural background of your patient

- More often a barrier
- More often an enabler
- Both a barrier and an enabler
- Neither a barrier or an enabler
- Not relevant to my practice

Q77 Support from / goals of the multi-disciplinary team or other clinicians treating your patients

- More often a barrier
- More often an enabler
- Both a barrier and an enabler
- Neither a barrier or an enabler
- Not relevant to my practice

Q78 Reason for dietitian referral

- More often a barrier
- More often an enabler
- Both a barrier and an enabler
- Neither a barrier or an enabler
- Not relevant to my practice

Q79 Time allocated to patient consultations

- More often a barrier
- More often an enabler
- Both a barrier and an enabler
- Neither a barrier or an enabler
- Not relevant to my practice

Q80 Number of patient visits

- More often a barrier
- More often an enabler
- Both a barrier and an enabler
- Neither a barrier or an enabler
- Not relevant to my practice

Q81 Cost of the diet

- Significant barrier
- More often an enabler
- Both a barrier and an enabler
- Neither a barrier or an enabler
- Not relevant to my practice

Q82 Can you think of any other barriers? *Please comment/ explain:*

Q83 Can you think of any other enablers? *Please comment/ explain:*

Display This Question:<br>If Q71 = More often an enabler<br>Or Q71 = Both a barrier and an enabler

Q84 You indicated that access to or awareness of professional development, education or training for dietitians is an enabler for you. *Can you please comment on what this includes:*

Display This Question:<br>If Q72 = More often an enabler<br>Or Q72 = Both a barrier and an enabler

Q85 You indicated that access to or awareness of relevant patient education materials or resources is an enabler for you. *Can you please comment on what this includes:*

^Q86 One of the key lifestyle related principles of the Mediterranean diet pattern is regular physical activity. The following questions relate to physical activity as part of your practice with the relevant chronic disease patient group/s

*Q87 Providing advice related to increasing regular physical activity to patients is part of my role

- ○ Strongly disagree
- ○ Somewhat disagree
- ○ Neither agree nor disagree
- ○ Somewhat agree
- ○ Strongly agree

*Q88 I am confident to provide advice related to increasing regular physical activity to my patients

- ○ Strongly disagree
- ○ Somewhat disagree
- ○ Neither agree nor disagree
- ○ Somewhat agree
- ○ Strongly agree

Q89 How often do you provide advice related to increasing regular physical activity to your patients?

- Never
- Rarely
- Sometimes
- Most of the time
- Always

*Q90 What advice would you typically provide? *Please comment/ explain*

*Q91 How often do you refer your patients to an exercise specialist?

- Never
- Rarely
- Sometimes
- Most of the time
- Always

*Q92 Would you estimate that you do an average of 150 minutes or more of moderate physical activity each week?

- Never
- Rarely
- Sometimes
- Most of the time
- Always

Q93 Are there any other comments you would like to add in relation to the questions asked in the survey? *Please comment/ explain*

^Description only. Not counted as a survey question item in tally as reported in manuscript
*Data not reported in this manuscript
